# The chromosomal genome sequence of the sponge *Crambe crambe *(Schmidt, 1862) and its associated microbial metagenome sequences

**DOI:** 10.12688/wellcomeopenres.24154.1

**Published:** 2025-05-23

**Authors:** Manuel Maldonado, Lucia Pita, Ute Hentschel, Dirk Erpenbeck, Graeme Oatley, Elizabeth Sinclair, Eerik Aunin, Noah Gettle, Camilla Santos, Michael Paulini, Haoyu Niu, Victoria McKenna, Rebecca O’Brien, Wellcome Sanger Institute Tree of Life Management, Samples and Laboratory Team, Wellcome Sanger Institute Scientific Operations: Sequencing Operations, Wellcome Sanger Institute Tree of Life Core Informatics Team, EBI Aquatic Symbiosis Genomics Data Portal Team, Aquatic Symbiosis Genomics Project Leadership

**Affiliations:** 1 Center for Advanced Studies of Blanes (CEAB-CSIC), Girona, Spain; 2 Institute of Marine Sciences – CSIC, Barcelona, Spain; 3 Integrated Marine Ecology group, Institute of Marine Research IIM-CSIC, Vigo, Spain; 4 GEOMAR Helmholtz Centre for Ocean Research Kiel, Kiel, Germany; 5 Ludwig-Maximilians University of Munich, Munich, Germany; 6 Tree of Life, Wellcome Sanger Institute, Hinxton, England, UK

**Keywords:** Crambe crambe, marine sponge, genome sequence, chromosomal, Poecilosclerida

## Abstract

We present a genome assembly from an individual *Crambe crambe* (Porifera; Demospongiae; Poecilosclerida; Crambeidae). The host genome sequence is 143.20 megabases in span. Most of the assembly is scaffolded into 18 chromosomal pseudomolecules. The mitochondrial genome has also been assembled and is 19.53 kilobases in length. Several symbiotic prokaryotic genomes were assembled as MAGs, including two relevant sponge symbionts, the *Candidatus* Beroebacter blanensis/ *AqS2* clade (Tethybacterales, Gammaproteobacteria) of LMA sponges, and the widely distributed archaeal *Nitrosopumilus* sp. clade.

## Species taxonomy

Eukaryota; Opisthokonta; Metazoa; Porifera; Demospongiae; Heteroscleromorpha; Poecilosclerida; Crambeidae; Crambe (in: sponges) *Crambe*; *Crambe crambe* Vosmaer, 1880 (NCBI:txid3722).

## Background

*Crambe crambe* ( Schmidt, 1862) is probably the most abundant sponge species in the sublittoral rocky bottoms of the Atlantic-Mediterranean region. It is a bright red encrusting sponge that grows at both well-lit and poorly lit sites, forming patches of up to 0.5 m ^2^ ( Pansini & Pronzato, 1990; Turon *et al.*, 1998). As additional macroscopic clues for species identification, oscula and their radially converging excurrent channels are often visible on the sponge surface, which is slippery to the touch. The sponge grows not only on rocks, but also on barnacles and on the shells of the red oyster *Spondylus gaederopus*.

Due to its abundance, the species is ecologically important in many ways. For instance, its skeletal growth represents a substantial silicon sink for the sublittoral system ( Maldonado *et al.*, 2005). The sponge also provides food and habitat for a variety of marine organisms, including recruitment habitat for juvenile ophiuroids ( Turon *et al.*, 2000) and small benthic fish. *C. crambe* produces various bioactive compounds that interact chemically with many community members ( Becerro *et al.*, 1994; Becerro *et al.*, 1997), some of which have potential pharmaceutical applications derived from their antibacterial, antifungal, and anti-tumour properties, among others ( El-Demerdash *et al.*, 2018). Given its biotechnological potential, attempts have been made to farm the species ( Padiglia *et al.*, 2018). Despite its abundance and ecological versatility (or perhaps because of it), the species is thought to be a surviving relict of the Jurassic oceans. This hypothesis is supported by the observation that the formation of all four spicule types is only possible at a silicate concentration ≥100 µM – concentrations which are likely to have occurred in Jurassic seas before the ecological expansion of diatoms ( Maldonado *et al.*, 1999). Secondly, the biogeographic distribution of the genus *Crambe* shows a clear Tethyan pattern ( Maldonado *et al.*, 2001).

Regarding the microbiome, the sponge is a species with low microbial abundance. While most of the few microbes occur in low abundance extracellularly in the mesohyl and around the skeletal spongin fibres, some of the microbes have been documented by electron microscopy to be contained within vesicles in the cytoplasm of bacteriocytes that appear to contain a single microbial species per cell ( Carrier *et al.*, 2022; Maldonado, 2007). Gammaproteobacteria, ammonia-oxidising *Nitrosopumilus* sp. (Archaea) and a single taxon, *Candidatus* Beroebacter blanensis, dominate the microbial community . This latter symbiont clade appears to be vertically transmitted ( Turon *et al.*, 2024). It was originally classified as *Betaproteobacteria* ( Croué *et al.*, 2013), but was later identified as *Ca.* Beroebacter blanensis, belonging to a novel bacterial order, *Candidatus* ( *Ca.*) Tethybacterales within the Gammaproteobacteria and consisting mainly of sponge symbionts ( Taylor *et al.*, 2021). The well characterized symbiont “AqS2” of *Amphimedon queenslandica* is the nearest phylogenetic relative of the *B. blanensis* clade, which displays genome reduction and limited metabolic capabilities, likely reflecting an adaptation to a symbiotic lifestyle within the sponge host ( Gauthier *et al.*, 2016).

The sexual condition of the species is hermaphroditism. It is worth noting that its spermatozoa are highly atypical within the phylum. They are very elongated and V-shaped, with the flagellum inserted in an antero-lateral position next to a true acrosome ( Riesgo & Maldonado, 2009; Tripepi *et al.*, 1984). This general organisation of the spermatozoon, which closely resembles that of Phoronida spermatozoa, appears to be common in the order Poecilosclerida but not in other sponges. Fertilisation is internal, and embryos are incubated for several months, until they develop into bright red, non-tufted parenchymella larvae ( Maldonado & Bergquist, 2002; Uriz *et al.*, 2001). In western Mediterranean populations, larval release extends from mid-July to mid-August, and larval production can be as high as 76 embryos per cm ^2 ^of sponge tissue ( Uriz *et al.*, 1998), which would explain the abundance of adults.

The sequencing of the whole-chromosome genome of *C. crambe* will facilitate in-depth understanding of the genomic basis of this species biology, as well as its ecology and evolution. This genome will be particularly useful for investigating the evolution of sexual strategies in Demospongiae, as well as for clarifying between-family relationships within the order Poecilosclerida. Together with the genome sequences of *C. crambe* microbial symbionts presented here, the novel data will enable targeted examination of the molecular basis of sponge silicate metabolism and skeleton formation, alkaloid metabolism, and sponge-microbe interactions in the role of carbon cycling, among other key questions in sponge symbiosis.

## Genome sequence report

**Figure 1.**
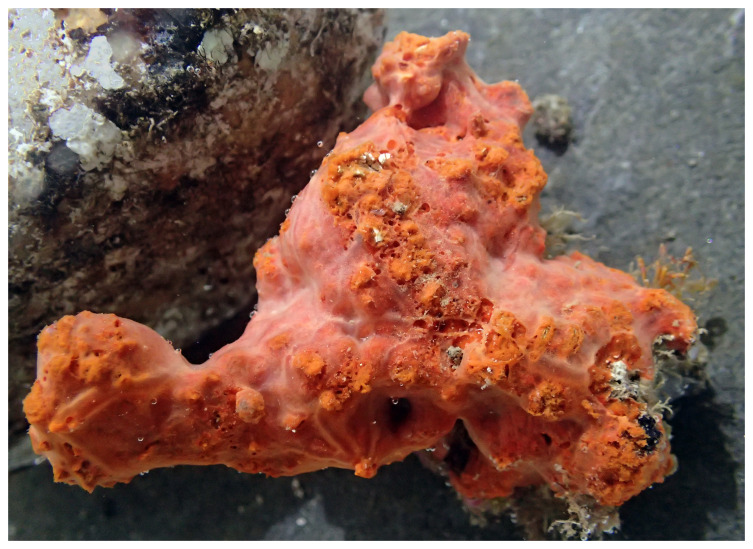
Photograph of the *Crambe crambe* (odCraCram1) specimen used for genome sequencing.

The genome was sequenced from an adult *Crambe crambe* ( Figure 1) collected from Blanes, Girona, Spain. A total of 459-fold coverage in Pacific Biosciences single-molecule HiFi long reads was generated. Primary assembly contigs were scaffolded with chromosome conformation Hi-C data. Manual assembly curation corrected 62 missing joins or mis-joins and removed 18 haplotypic duplications, reducing the assembly length by 2.19% and the scaffold number by 29.78%, also decreasing the scaffold N50 by 0.31%.

**Figure 2.**
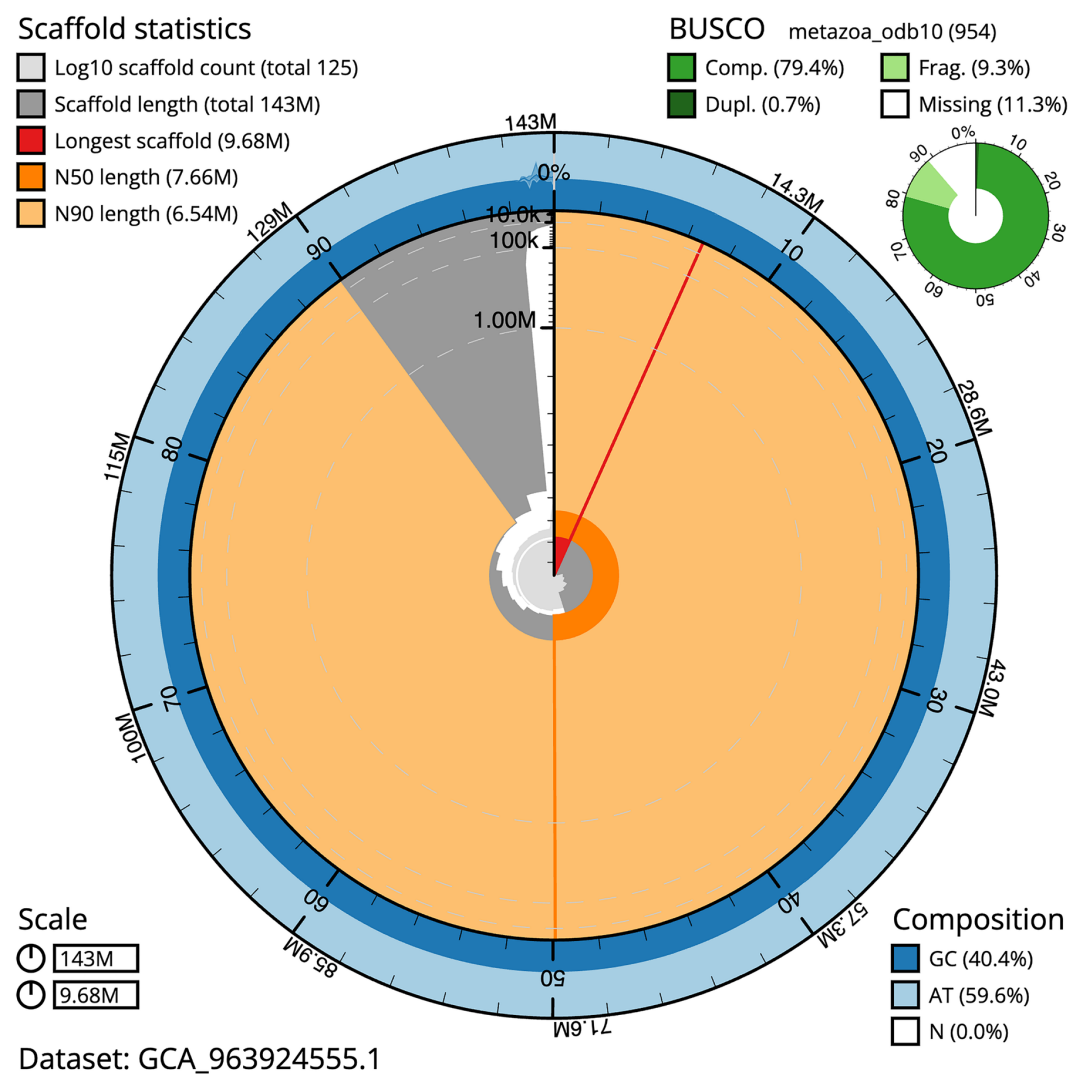
Genome assembly of *Crambe crambe*, odCraCram1.1: metrics. The BlobToolKit Snailplot shows N50 metrics and BUSCO gene completeness. The main plot is divided into 1,000 size-ordered bins around the circumference with each bin representing 0.1% of the 143,197,480 bp assembly. The distribution of scaffold lengths is shown in dark grey with the plot radius scaled to the longest scaffold present in the assembly (9,683,886 bp, shown in red). Orange and pale-orange arcs show the N50 and N90 scaffold lengths (7,656,483 and 6,535,638 bp), respectively. The pale grey spiral shows the cumulative scaffold count on a log scale with white scale lines showing successive orders of magnitude. The blue and pale-blue area around the outside of the plot shows the distribution of GC, AT and N percentages in the same bins as the inner plot. A summary of complete, fragmented, duplicated and missing BUSCO genes in the metazoa_odb10 set is shown in the top right. An interactive version of this figure is available at https://blobtoolkit.genomehubs.org/view/Crambe_crambe/dataset/GCA_963924555.1/snail.

**Figure 3.**
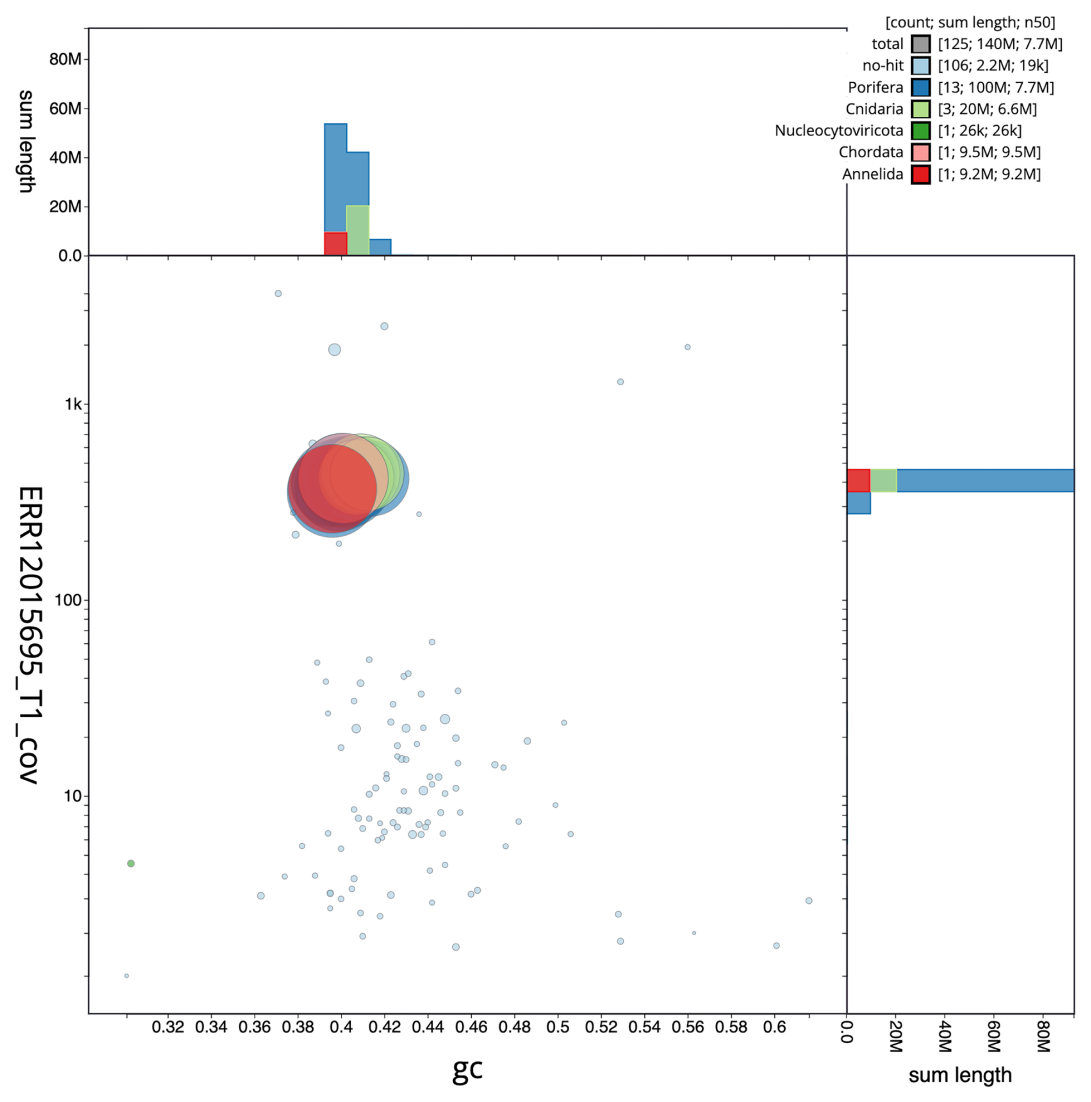
Genome assembly of *Crambe crambe*, odCraCram1.1: BlobToolKit GC-coverage plot. Scaffolds are coloured by phylum. Circles are sized in proportion to scaffold length. Histograms show the distribution of scaffold length sum along each axis. An interactive version of this figure is available at https://blobtoolkit.genomehubs.org/view/Crambe_crambe/dataset/GCA_963924555.1/blob.

**Figure 4.**
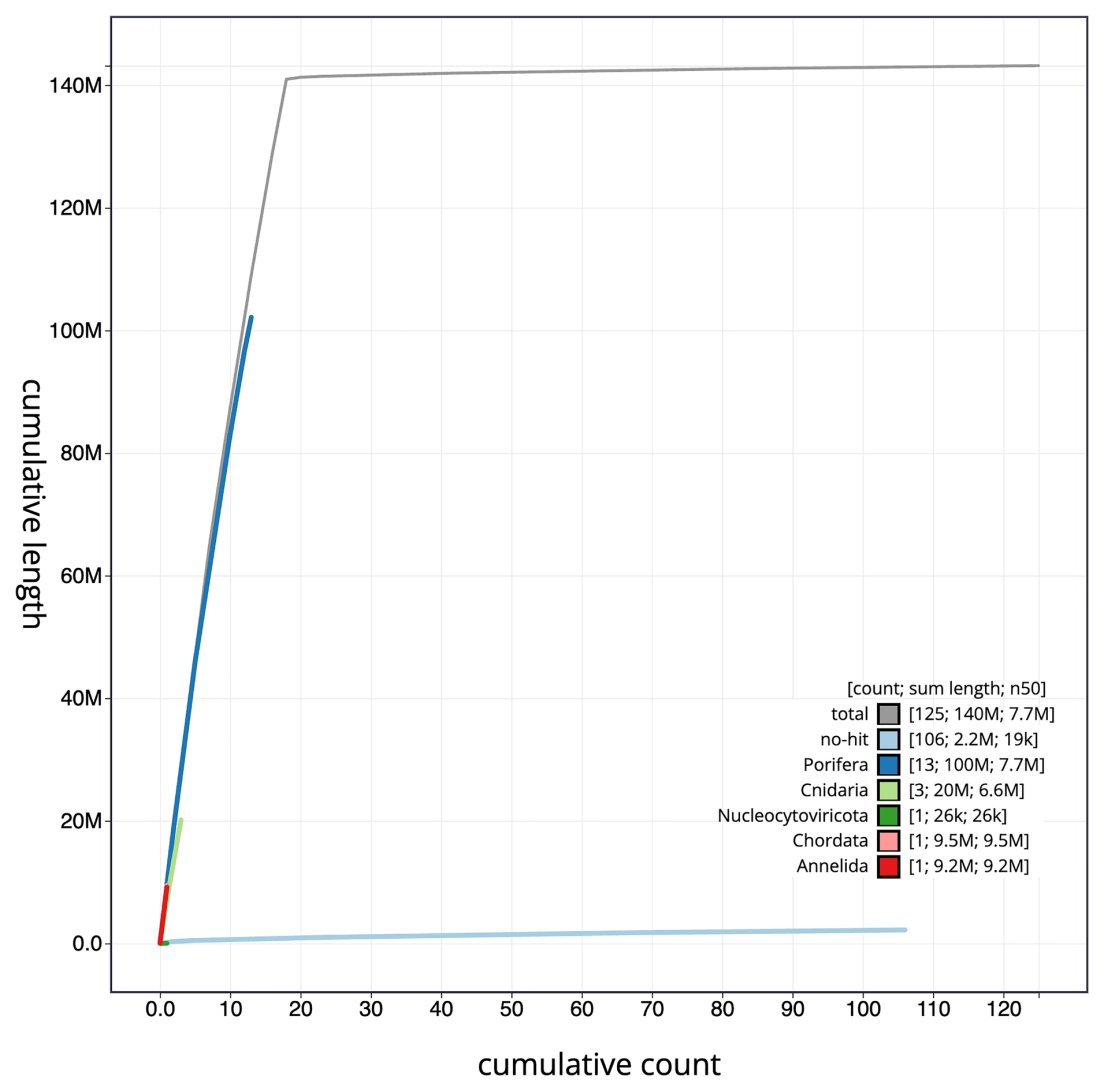
Genome assembly of *Crambe crambe*, odCraCram1.1: BlobToolKit cumulative sequence plot. The grey line shows cumulative length for all scaffolds. Coloured lines show cumulative lengths of scaffolds assigned to each phylum using the buscogenes taxrule. An interactive version of this figure is available at https://blobtoolkit.genomehubs.org/view/Crambe_crambe/dataset/GCA_963924555.1/cumulative.

**Figure 5.**
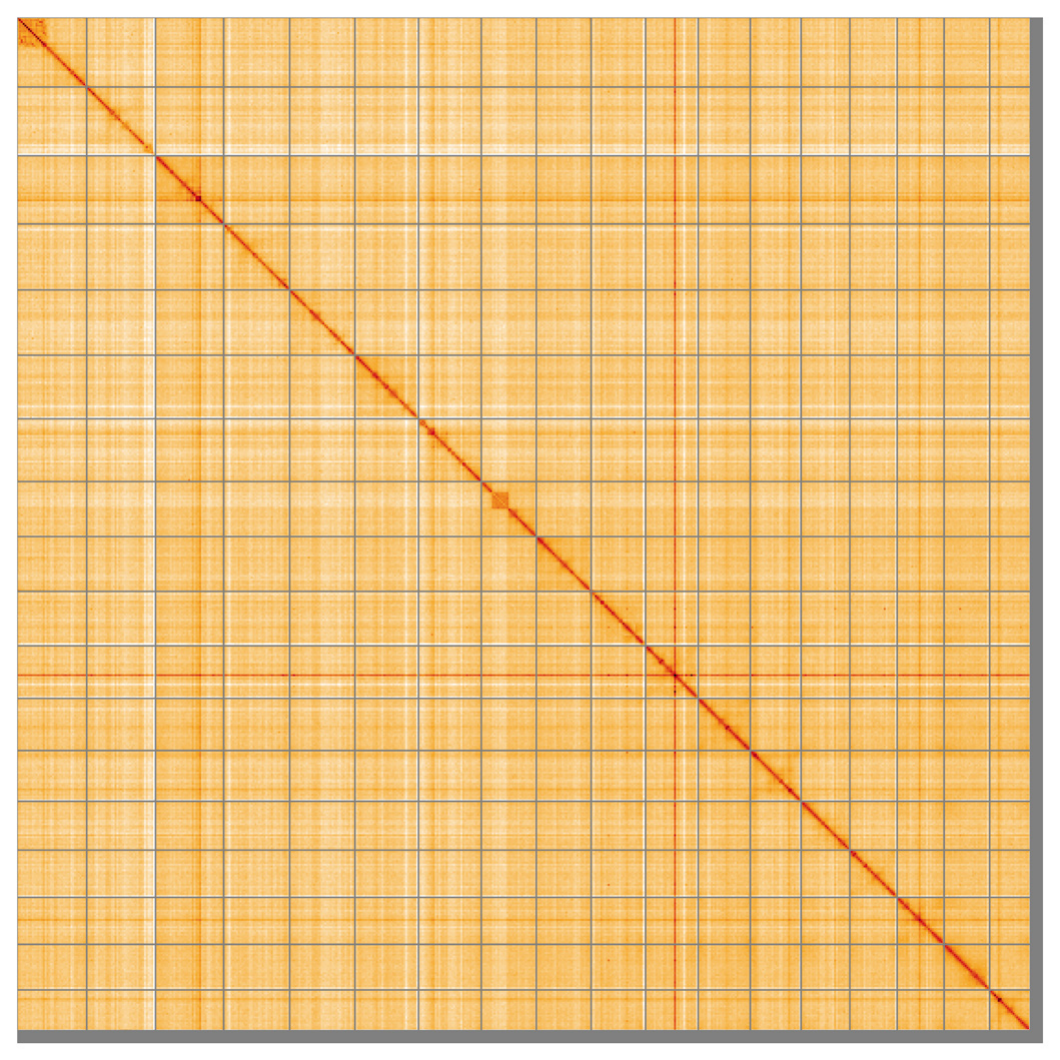
Genome assembly of *Crambe crambe*, odCraCram1.1: Hi-C contact map of the odCraCram1.1 assembly, visualised using HiGlass. Chromosomes are shown in order of size from left to right and top to bottom. An interactive version of this figure may be viewed at https://genome-note-higlass.tol.sanger.ac.uk/l/?d=IeGb4iyXTOqWeiUVkpdMrA.

The final assembly has a total length of 143.20 Mb in 124 sequence scaffolds with a scaffold N50 of 7.7 Mb ( Table 1). The snail plot in Figure 2 provides a summary of the assembly statistics, while the distribution of assembly scaffolds on GC proportion and coverage is shown in Figure 3. The cumulative assembly plot in Figure 4 shows curves for subsets of scaffolds assigned to different phyla. Most (98.69%) of the assembly sequence was assigned to 18 chromosomal-level scaffolds. Chromosome-scale scaffolds confirmed by the Hi-C data are named in order of size ( Figure 5; Table 2). While not fully phased, the assembly deposited is of one haplotype. Contigs corresponding to the second haplotype have also been deposited. The mitochondrial genome was also assembled and can be found as a contig within the multifasta file of the genome submission.

**Table 1.** Genome data for *Crambe crambe*, odCraCram1.1.

Project accession data			
Assembly identifier	odCraCram1.1		
Species	*Crambe crambe*		
Specimen	odCraCram1		
NCBI taxonomy ID	3722		
BioProject	PRJEB65618		
BioSample ID	Genome sequencing: SAMEA9361910 Hi-C scaffolding: SAMEA9361908		
Isolate information	odCraCram1: (genome and Hi-C sequencing)		
Assembly metrics			
Consensus quality (QV)	58.1		
BUSCO *	C:78.8%[S:78.0%,D:0.8%],F:9.4%,M:11.8%,n:954		
Percentage of assembly mapped to chromosomes	98.69%		
Organelles	Mitochondrial genome: 19.53 kb		
Sequencing information			
**Platform**	**Run accession**	**Read count**	**Base count (Gb)**
**Hi-C Illumina NovaSeq 6000**	ERR12512721	1.13e+09	170.77
**PacBio Revio**	ERR12015695	9.82e+06	67.94
Genome assembly			
Assembly accession	GCA_963924555.1		
*Accession of alternate haplotype*	GCA_963924525.1		
Span (Mb)	143.20		
Number of contigs	178		
Contig N50 length (Mb)	3.5		
Number of scaffolds	124		
Scaffold N50 length (Mb)	7.7		
Longest scaffold (Mb)	9.77		

* BUSCO scores based on the metazoa_odb10 BUSCO set using version 5.4.3. C = complete [S = single copy, D = duplicated], F = fragmented, M = missing, n = number of orthologues in comparison. A full set of BUSCO scores is available at https://blobtoolkit.genomehubs.org/view/Crambe_crambe/dataset/GCA_963924555.1/busco.

**Table 2.** Chromosomal pseudomolecules in the genome assembly of *Crambe crambe*, odCraCram1.

INSDC accession	Name	Length (Mb)	GC%
OZ004581.1	1	9.57	39.5
OZ004582.1	2	9.68	40.0
OZ004583.1	3	9.49	40.0
OZ004584.1	4	9.08	40.0
OZ004585.1	5	9.18	39.5
OZ004586.1	6	8.84	40.0
OZ004587.1	7	8.73	39.5
OZ004588.1	8	7.66	40.0
OZ004589.1	9	7.63	40.5
OZ004590.1	10	7.57	40.5
OZ004591.1	11	7.33	40.5
OZ004592.1	12	7.24	41.0
OZ004593.1	13	7.06	40.5
OZ004594.1	14	6.8	40.5
OZ004595.1	15	6.57	40.5
OZ004596.1	16	6.54	41.5
OZ004597.1	17	6.34	41.0
OZ004598.1	18	5.67	40.5
OZ004599.1	MT	0.02	37.0

The estimated Quality Value (QV) of the final assembly is 58.1. The assembly has a BUSCO v5.4.3 completeness of 78.8% (single = 78.0%, duplicated = 0.8%), using the metazoa_odb10 reference set ( *n* = 954).

## Metagenome report

**Figure 6.**
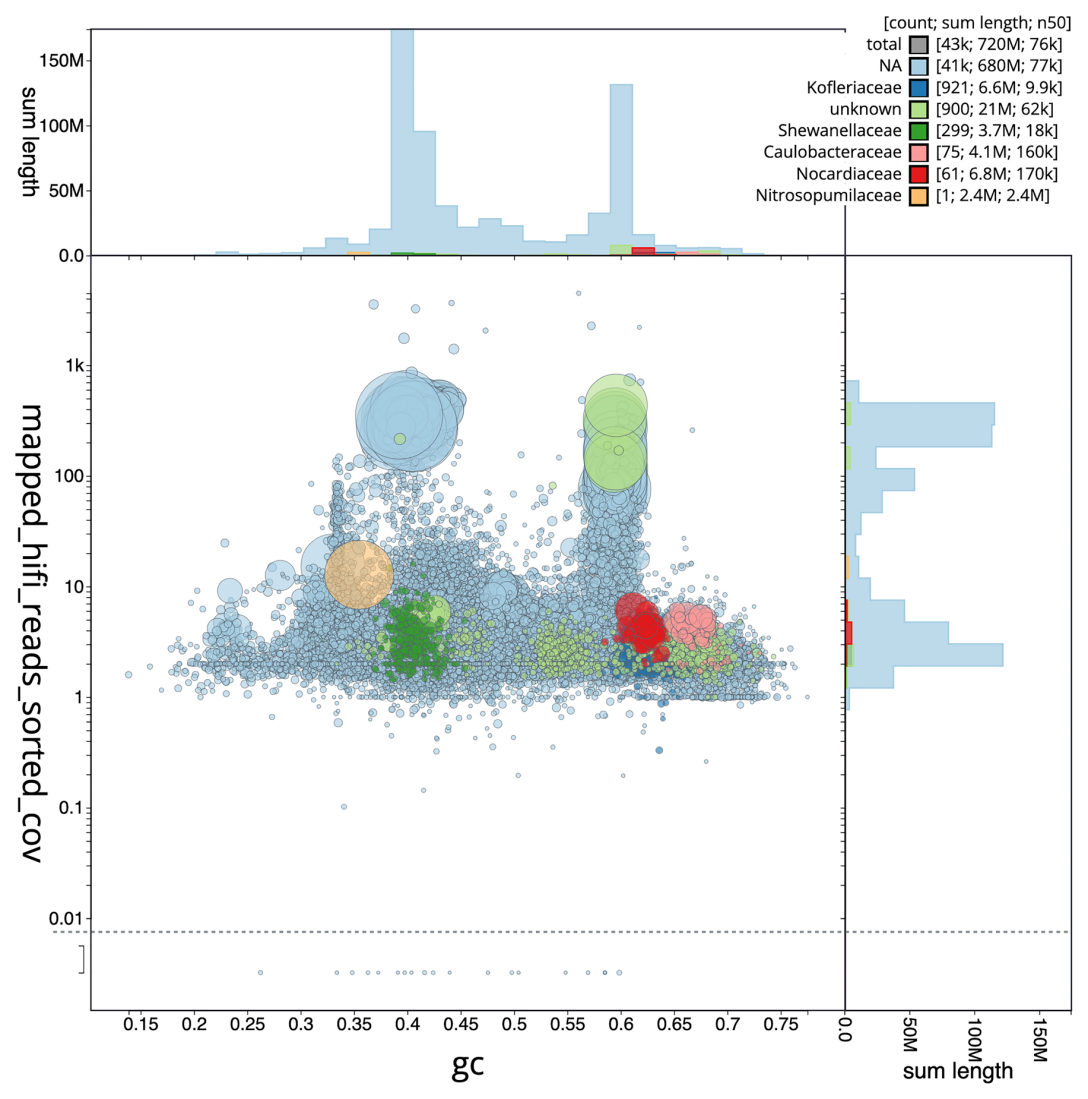
Blob plot of base coverage in mapped against GC proportion for sequences in the metagenome of *Crambe crambe*. Binned metagenomes are coloured by family. Circles are sized in proportion to sequence length on a square root scale, ranging from 501 to 4,126,685. Histograms show the distribution of sequence length sum along each axis An interactive version of this figure may be viewed here.

**Figure 7.**
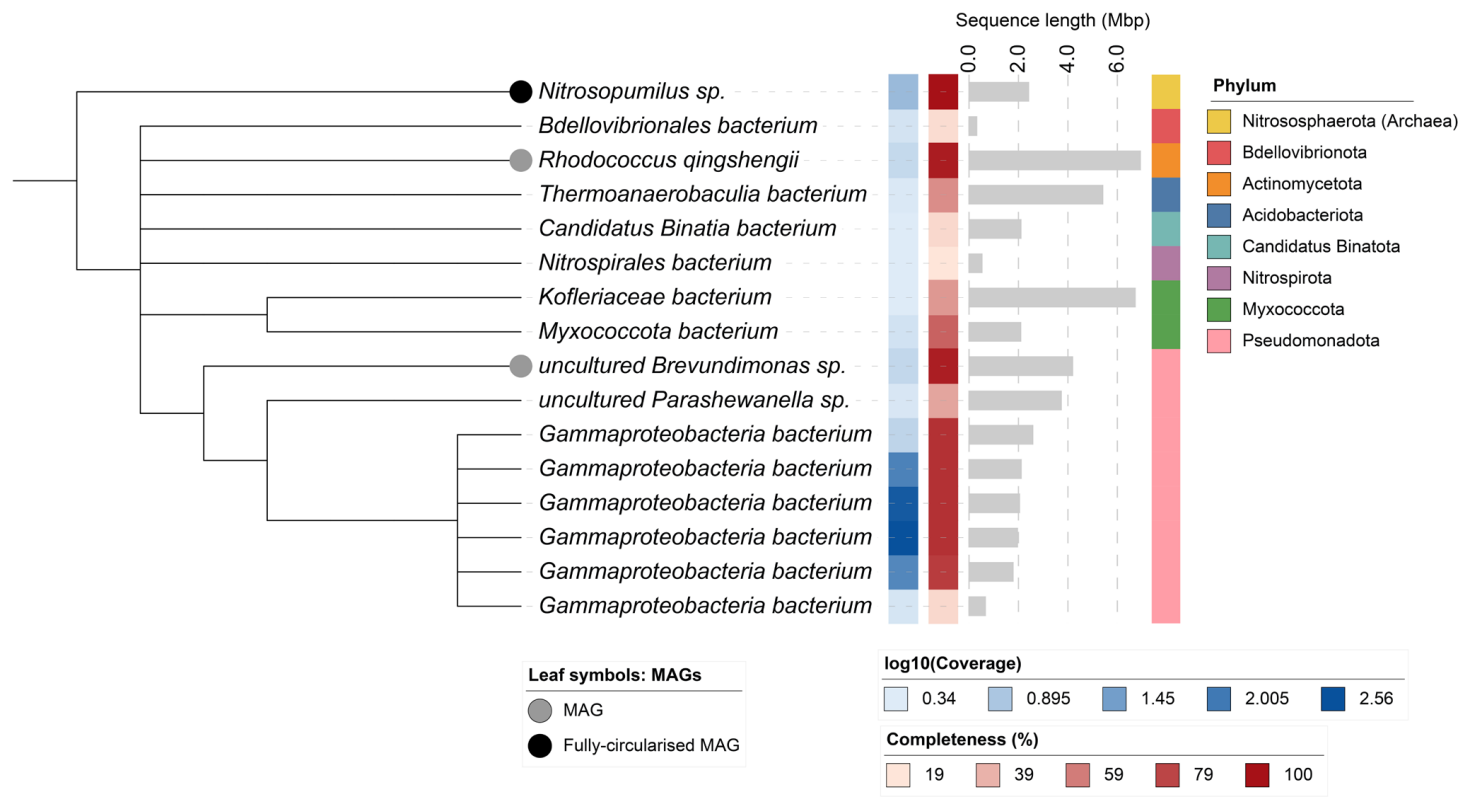
Cladogram showing the taxonomic placement of metagenome bins, constructed using NCBI taxonomic identifiers with *taxonomizr* and annotated in iTOL. Colours indicate phylum-level taxonomy. Additional tracks show sequencing coverage (log₁₀), estimated genome size (Mbp), and completeness. Bins that meet the criteria for MAGs are marked with a grey circle; the single fully circularised MAG is marked in black.

Sixteen binned genomes were generated from the metagenome assembly ( Figure 6), of which three were classified as high-quality metagenome assembled genomes (MAGs) (see methods). The completeness values for these assemblies range from approximately 20% to 100% with contamination below 7%. A cladogram of the binned metagenomes is shown in Figure 7. For details on binned genomes see Table 3.

**Table 3.** Quality metrics and taxonomic assignments of the binned metagenomes.

NCBI taxon	Taxid	GTDB taxonomy	Quality	Size (bp)	Contigs	Circular	Mean coverage	Completeness (%)	Contamination (%)
Nitrosopumilus sp.	2024843	g__Nitrosopumilus	High	2,406,465	1	Yes	11.72	100.00	0.00
uncultured Brevundimonas sp.	213418	g__Brevundimonas	High	4,185,465	75	Partial	4.39	95.06	4.27
Rhodococcus qingshengii	334542	s__Rhodococcus qingshengii	High	6,934,702	61	No	4.2	95.58	0.00
Gammaproteobacteria bacterium	1913989	f__AqS2	Medium	1,777,560	2	No	61.05	83.22	1.22
Gammaproteobacteria bacterium	1913989	f__AqS2	Medium	1,964,849	1	Yes	364.77	87.49	0.61
Gammaproteobacteria bacterium	1913989	f__AqS2	Medium	2,039,060	1	Yes	278.56	87.49	0.61
Myxococcota bacterium	2818507	f__UBA6930	Medium	2,090,974	133	No	3.11	69.12	6.72
Gammaproteobacteria bacterium	1913989	f__AqS2	Medium	2,109,981	4	No	74.99	87.49	1.83
Gammaproteobacteria bacterium	1913989	g__UBA1858	Medium	2,576,596	26	No	4.79	87.52	1.97
Thermoanaerobaculia bacterium	2651171	f__UBA5704	Medium	5,408,624	319	No	2.54	52.97	1.44
Bdellovibrionales bacterium	2053517	g__JACOND01	Low	314,367	16	No	2.89	23.10	0.00
Nitrospirales bacterium	2358460	g__Bin75	Low	524,459	58	No	2.22	19.47	0.00
Gammaproteobacteria bacterium	1913989	g__UBA1858	Low	655,358	63	No	2.67	24.99%	1.77%
Candidatus Binatia bacterium	2838779	g__JAAXHF01	Low	2,103,514	277	No	2.23	24.60%	0.00%
uncultured Parashewanella sp.	2547967	g__Parashewanella	Low	3,728,522	299	Partial	2.61	43.78%	5.25%
Kofleriaceae bacterium	2212474	f__Haliangiaceae	Low	6,724,497	921	Partial	2.17	48.48%	5.11%

## Methods

### Sample acquisition

A specimen of *Crambe crambe* (specimen ID GHC0000181, ToLID odCraCram1) was collected from Blanes, Girona, Spain (latitude 41.67, longitude 2.80) on 2021-02-01 by SCUBA diving. The specimen was collected and identified by Manuel Maldonado (CEAB-CSIC) and preserved by snap-freezing.

### Nucleic acid extraction

The workflow for high molecular weight (HMW) DNA extraction at the Wellcome Sanger Institute (WSI) Tree of Life Core Laboratory includes a sequence of core procedures: sample preparation; sample homogenisation, DNA extraction, fragmentation, and clean-up. Protocols are available on protocols.io ( Denton *et al.*, 2023). In sample preparation, the odCraCram1 sample was weighed and dissected on dry ice ( Jay *et al.*, 2023). Prior to DNA extraction, the sponge sample was bathed in “L buffer” (10 mM Tris, pH 7.6, 100 mM EDTA, 20 mM NaCl), minced into small pieces using a scalpel and the cellular interior separated from the mesohyl using forceps ( Lopez, 2022). HMW DNA was extracted using the Manual MagAttract v1 protocol ( Strickland *et al.*, 2023b). DNA was sheared into an average fragment size of 12–20 kb in a Megaruptor 3 system ( Todorovic *et al.*, 2023). Sheared DNA was purified by solid-phase reversible immobilisation ( Strickland *et al.*, 2023a), using AMPure PB beads to eliminate shorter fragments and concentrate the DNA. The concentration of the sheared and purified DNA was assessed using a Nanodrop spectrophotometer, Qubit Fluorometer and Qubit dsDNA High Sensitivity Assay kit. Fragment size distribution was evaluated by running the sample on the FemtoPulse system.

### Sequencing

Pacific Biosciences HiFi circular consensus DNA sequencing libraries were constructed according to the manufacturers’ instructions. DNA sequencing was performed by the Scientific Operations core at the WSI on a Pacific Biosciences Revio instrument. Hi-C data were also generated from tissue of odCraCram1 using the Arima2 kit and sequenced on the Illumina NovaSeq 6000 instrument.

### Host genome assembly and curation

Assembly was carried out with Hifiasm ( Cheng *et al.*, 2021) and haplotypic duplication was identified and removed with purge_dups ( Guan *et al.*, 2020). The assembly was then scaffolded with Hi-C data ( Rao *et al.*, 2014) using YaHS ( Zhou *et al.*, 2023). The mitochondrial genome was assembled using MitoHiFi ( Uliano-Silva *et al.*, 2023), which runs MitoFinder ( Allio *et al.*, 2020) and uses these annotations to select the final mitochondrial contig and to ensure the general quality of the sequence. Table 4 contains a list of relevant software tool versions and sources.

**Table 4.** Software tools: versions and sources.

Software tool	Version	Source
BEDTools	2.30.0	https://github.com/arq5x/bedtools2
bin3C	0.3.3	https://github.com/cerebis/bin3C
Blast	2.14.0	ftp://ftp.ncbi.nlm.nih.gov/blast/executables/blast+/
BlobToolKit	4.3.7	https://github.com/blobtoolkit/blobtoolkit
BUSCO	5.4.3 and 5.5.0	https://gitlab.com/ezlab/busco
bwa-mem2	2.2.1	https://github.com/bwa-mem2/bwa-mem2
CheckM	1.2.1	https://github.com/Ecogenomics/CheckM
Cooler	0.8.11	https://github.com/open2c/cooler
DAS Tool	-	https://github.com/cmks/DAS_Tool
DIAMOND	2.1.8	https://github.com/bbuchfink/diamond
dRep	3.4.0	https://github.com/MrOlm/drep
fasta_windows	0.2.4	https://github.com/tolkit/fasta_windows
FastK	427104ea91c78c3b8b8b49f1a7d6bbeaa869ba1c	https://github.com/thegenemyers/FASTK
GoaT CLI	0.2.5	https://github.com/genomehubs/goat-cli
GTDB-TK	2.3.2	https://github.com/Ecogenomics/GTDBTk
Hifiasm	0.19.5-r587	https://github.com/chhylp123/hifiasm
HiGlass	44086069ee7d4d3f6f3f0012569789ec138f42b84 aa44357826c0b6753eb28de	https://github.com/higlass/higlass
MaxBin	2.7	https://sourceforge.net/projects/maxbin/
MerquryFK	d00d98157618f4e8d1a9190026b19b471055b22e	https://github.com/thegenemyers/MERQURY.FK
MetaBat2	2.15-15-gd6ea400	https://bitbucket.org/berkeleylab/metabat/src/master/
MetaTOR	-	https://github.com/koszullab/metaTOR
MitoHiFi	2	https://github.com/marcelauliano/MitoHiFi
MultiQC	1.14, 1.17, and 1.18	https://github.com/MultiQC/MultiQC
Nextflow	23.04.0-5857	https://github.com/nextflow-io/nextflow
PretextView	0.2	https://github.com/wtsi-hpag/PretextView
PROKKA	1.14.5	https://github.com/vdejager/prokka
purge_dups	1.2.5	https://github.com/dfguan/purge_dups
samtools	1.16.1, 1.17, and 1.18	https://github.com/samtools/samtools
Seqtk	1.3	https://github.com/lh3/seqtk
Singularity	3.9.0	https://github.com/sylabs/singularity
TreeVal	1.0.0	https://github.com/sanger-tol/treeval
YaHS	1.1a.2	https://github.com/c-zhou/yahs

The assembly was checked for contamination and corrected using the TreeVal pipeline ( Pointon *et al.*, 2023). Manual curation was primarily conducted using PretextView ( Harry, 2022), with additional insights provided by JBrowse2 ( Diesh *et al.*, 2023) and HiGlass ( Kerpedjiev *et al.*, 2018). Any identified contamination, missed joins, and mis-joins were corrected, and duplicate sequences were tagged and removed. The curation process is documented at https://gitlab.com/wtsi-grit/rapid-curation.

### Taxonomic verification

Molecular markers obtained from the assembly were used to reconstruct the phylogenetic position of the sample. In an alignment using MAFFT v7.450 ( Katoh & Standley, 2013), the COI barcoding fragment (“Folmer” fragment) of the sample was found to be identical to haplotype 1 from a dedicated study on *Crambe crambe* ( Duran *et al.*, 2004, AF526297), besides samples from other studies on this species as published in NCBI Genbank.

### Host assembly quality assessment

The Merqury.FK tool ( Rhie *et al.*, 2020), run in a Singularity container ( Kurtzer *et al.*, 2017), was used to evaluate *k*-mer completeness and assembly quality for the primary and alternate haplotypes using the *k*-mer databases ( *k* = 31) that were computed prior to genome assembly. The analysis outputs included assembly QV scores and completeness statistics.

A Hi-C contact map was produced for the final version of the assembly. The Hi-C reads were aligned using bwa-mem2 ( Vasimuddin *et al.*, 2019) and the alignment files were combined using SAMtools ( Danecek *et al.*, 2021). The Hi-C alignments were converted into a contact map using BEDTools ( Quinlan & Hall, 2010) and the Cooler tool suite ( Abdennur & Mirny, 2020). The contact map is visualised in HiGlass ( Kerpedjiev *et al.*, 2018).

The blobtoolkit pipeline is a Nextflow port of the previous Snakemake Blobtoolkit pipeline ( Challis *et al.*, 2020). It aligns the PacBio reads in SAMtools and minimap2 ( Li, 2018) and generates coverage tracks for regions of fixed size. In parallel, it queries the GoaT database ( Challis *et al.*, 2023) to identify all matching BUSCO lineages to run BUSCO ( Manni *et al.*, 2021). For the three domain-level BUSCO lineages, the pipeline aligns the BUSCO genes to the UniProt Reference Proteomes database ( Bateman *et al.*, 2023) with DIAMOND blastp ( Buchfink *et al.*, 2021). The genome is also divided into chunks according to the density of the BUSCO genes from the closest taxonomic lineage, and each chunk is aligned to the UniProt Reference Proteomes database using DIAMOND blastx. Genome sequences without a hit are chunked using seqtk and aligned to the NT database with blastn ( Altschul *et al.*, 1990). The blobtools suite combines all these outputs into a blobdir for visualisation.

The blobtoolkit pipeline was developed using nf-core tooling ( Ewels *et al.*, 2020) and MultiQC ( Ewels *et al.*, 2016), relying on the Conda package manager, the Bioconda initiative ( Grüning *et al.*, 2018), the Biocontainers infrastructure ( da Veiga Leprevost *et al.*, 2017), as well as the Docker ( Merkel, 2014) and Singularity ( Kurtzer *et al.*, 2017) containerisation solutions.

### Metagenome assembly

The metagenome assembly was generated using metaMDBG ( Benoit *et al.*, 2024) and binned using MetaBAT2 ( Kang *et al.*, 2019), MaxBin ( Wu *et al.*, 2014), bin3C ( DeMaere & Darling, 2019), and MetaTOR. The resulting bin sets of each binning algorithm were optimised and refined using DAS Tool ( Sieber *et al.*, 2018). PROKKA ( Seemann, 2014) was used to identify tRNAs and rRNAs in each bin, CheckM ( Parks *et al.*, 2015) (checkM_DB release 2015-01-16) was used to assess bin completeness/contamination, and GTDB-TK ( Chaumeil *et al.*, 2022) (GTDB release 214) was used to taxonomically classify bins. Taxonomic replicate bins were identified using dRep ( Olm *et al.*, 2017), with default settings (95% ANI threshold). The final bin set was filtered for bacteria and archaea. All bins were assessed for quality and categorised as metagenome-assembled genomes (MAGs) if they met the following criteria: contamination ≤ 5%, presence of 5S, 16S, and 23S rRNA genes, at least 18 unique tRNAs, and either ≥ 90% completeness or ≥ 50% completeness with fully circularised chromosomes. Bins that did not meet these thresholds, or were identified as taxonomic replicates of MAGs, were retained as ‘binned metagenomes’ provided they had ≥ 50% completeness and ≤ 10% contamination. A cladogram based on NCBI taxonomic assignments was generated using the ‘taxonomizr’ package in R. The tree was visualised and annotated using iTOL ( Letunic & Bork, 2024). Software tool versions and sources are given in Table 4.

### Wellcome Sanger Institute – Legal and Governance

The materials that have contributed to this genome note have been supplied by a Tree of Life collaborator. The Wellcome Sanger Institute employs a process whereby due diligence is carried out proportionate to the nature of the materials themselves, and the circumstances under which they have been/are to be collected and provided for use. The purpose of this is to address and mitigate any potential legal and/or ethical implications of receipt and use of the materials as part of the research project, and to ensure that in doing so we align with best practice wherever possible. The overarching areas of consideration are:

• Ethical review of provenance and sourcing of the material

• Legality of collection, transfer and use (national and international)

Each transfer of samples is undertaken according to a Research Collaboration Agreement or Material Transfer Agreement entered into by the Tree of Life collaborator, Genome Research Limited (operating as the Wellcome Sanger Institute) and in some circumstances other Tree of Life collaborators.

## Data Availability

European Nucleotide Archive: *Crambe crambe*. Accession number PRJEB65618; https://identifiers.org/ena.embl/PRJEB65618. The genome sequence is released openly for reuse. The *Crambe crambe* genome sequencing initiative is part of the Aquatic Symbiosis Genomics (ASG) project ( https://www.ebi.ac.uk/ena/browser/view/PRJEB43743). All raw sequence data and the assembly have been deposited in INSDC databases. The genome will be annotated using available RNA-Seq data and presented through the Ensembl pipeline at the European Bioinformatics Institute. Raw data and assembly accession identifiers are reported in Table 1 and Table 2.

## Author information

Members of the Wellcome Sanger Institute Tree of Life Management, Samples and Laboratory Team are listed here: https://doi.org/10.5281/zenodo.10066175.

Members of the Wellcome Sanger Institute Scientific Operations: Sequencing Operations are listed here: https://doi.org/10.5281/zenodo.10043364.

Members of the Wellcome Sanger Institute Tree of Life Core Informatics team are listed here: https://doi.org/10.5281/zenodo.10066637.

Members of the European Bioinformatics Institute ASG Data Portal team are listed here: https://doi.org//10.5281/zenodo.10076466.

Members of the Wellcome Sanger Institute/Aquatic Symbiosis Genomics Project Leadership are listed here: https://doi.org/10.5281/zenodo.10184833.
