# Supplementary material for: The chromosomal genome sequence of the sponge Crambe crambe (Schmidt, 1862) and its associated microbial metagenome sequences
Source: Wellcome Open Res. 2025 May 23;10:275. [Version 1] doi: 10.12688/wellcomeopenres.24154.1 (PMC12166358; doi:10.12688/wellcomeopenres.24154.1)
Supplement: Peer review history

# Reviewer report: Reviewer response for version 1

**DOI:** 10.21956/wellcomeopenres.26645.r124356

**Published:** 2025-06-13

**Authors:** Paco Cardenas

**Affiliations:** 1 Museum of Evolution, Uppsala University, Uppsala, Sweden

This article describes how the whole genome of the marine sponge *Crambe crambe* was sequenced and assembled into 18 chromosomes, along with several MAGs.

 Being a sponge systematist and not a genomics bioinformatician, I could only judge the biological part of the article, not the genomics and metagenomics technical parts.

 In the background, the authors mention that this species is a surviving relict. They give two arguments but miss a third important one, the one given by time-calibrated phylogenies suggesting the emergence of the order Poecilosclerida around the Triassic (Plese et al., 2021) (Ref 1).

 In Figure 1, a scale is missing.

Are sufficient details of methods and materials provided to allow replication by others?

Yes

Is the rationale for creating the dataset(s) clearly described?

Yes

Are the datasets clearly presented in a useable and accessible format?

Yes

Are the protocols appropriate and is the work technically sound?

Yes

Reviewer Expertise:

Porifera systematics and biology, Deep-sea habitats, Museum collections, Historical collections.

I confirm that I have read this submission and believe that I have an appropriate level of expertise to confirm that it is of an acceptable scientific standard.

## References

[1] : Mitochondrial evolution in the Demospongiae (Porifera): Phylogeny, divergence time, and genome biology. *Molecular Phylogenetics and Evolution* .2021;155: 10.1016/j.ympev.2020.107011 10.1016/j.ympev.2020.107011 33217579

# Reviewer report: Reviewer response for version 1

**DOI:** 10.21956/wellcomeopenres.26645.r124358

**Published:** 2025-06-12

**Authors:** Weizhi Song, Sihan Li

**Affiliations:** 1 The Hong Kong University of Science and Technology, Clear Water Bay, Hong Kong; 2 The Hong Kong University of Science and Technology Department of Ocean Science, Hong Kong, Hong Kong

This article presents the chromosomal genome sequence of the sponge *Crambe crambe* (Schmidt, 1862) and its associated microbial metagenome sequences. *Crambe crambe* is abundant in the sublittoral rocky bottoms of the Atlantic-Mediterranean region, and its genome (including that of its symbionts) was sequenced as part of the Aquatic Symbiosis Genomics Project. Cutting-edge sequencing technologies, including PacBio HiFi and Hi-C sequencing, were employed to generate the genome assemblies. Technical details for generating the assemblies are provided, and the resulting data can be publicly accessed via the European Nucleotide Archive. This dataset serves as a valuable resource for sponge-related research, ranging from investigations into sponge host phylogeny to studies on sponge-microbe symbiosis. However, several aspects require improvement before it can be accepted for publication. Please see details below.

 The units used for sequence length or assembly size are inconsistent throughout the article. For example, “Gb” and “Mb” are used in Tables 1 and 2; “M” and “k” appear in Figures 2, 3, 4, and 6; while “bp” and “Mbp” are used in Figure 7. Please ensure consistent format of length units in the article.

 Most of the figures (Figures 2 to 7) in this article appear to have been automatically generated, with little refinement for publication quality. Some of them are not informative and should be removed from the article. Specifically:

 Figure 2: It is not informative and should be removed from the article, the relevant statistical details are already clearly provided in figure legends. In addition, it is redundant to Table 1 in several aspects, including reports on BUSCO assessments, Scaffold N50, and Longest scaffold. Furthermore, there are inconsistencies between the statistics reported in Table 1 and those shown in Figure 1. For instance, the BUSCO completeness is listed as 78.8% in Table 1, but 79.4% in Figure 1. Please double check on these details.

 Figure 3: The “sum length” on both the x- and y-axes doesn’t make much sense and better to be removed from the figure.

 Figure 4: It is not informative, including a link to the plot within the article should be sufficient.

 Figure 5: Please enlarge it for better visibility. It appears that there may be issues with chromosomes 3, 8, and 11 (from left to right) (By the way, I couldn’t find an option to attach figures in this online review system to illustrate the issues I’ve identified). Could this be further refined or discussed in the main text?

 Figure 6: Similar to Figure 3, the “sum length” panels are meaningless, especially for the microbial metagenome, and should be removed from the figure. Also, the link to Figure 6 is not accessible.

 Figure 7: “Sequence length (Mbp)” in should be revised to “Genome Size (Mbp)”.

 Figure 7: I recommend building a phylogenetic tree for the generated MAGs, rather than a cladogram with lots of polytomies.

 Figure 7: Many of the leaves on the tree share the same name (e.g., *Gammaproteobacteria bacterium*). Please assign a unique identifier to each, such as the MAG name or accession number.

 Figure 7: Using a black symbol to indicate that a MAG is circularized is fine. However, the meaning of the two grey symbols is unclear to me, as all of them appear to be MAGs. If the intention is to distinguish between high-, medium-, or low-quality MAGs, please clarify that explicitly.

 Table 3: Some values in the “Completeness” and “Contamination” columns include a “%” symbol, while others do not. Please ensure consistency in formatting throughout the table.

 Table 4: there is no need to retain Table 4. Please ensure that all software tools used in data analysis, along with their versions, are properly cited in the “Methods” section. I noticed that software version is missing from the main text.

 “Metagenome assembly” section:

 GTDB release r214 came out in 2023 (two years ago). It may be worth considering reclassifying these MAGs using the most recent GTDB release (e.g., r226).

 “Metagenome assembly” section:

 “All bins were assessed for quality and categorised as metagenome-assembled genomes (MAGs) if they met the following criteria: …”. Is there a reference supporting the criteria used here?

Are sufficient details of methods and materials provided to allow replication by others?

Yes

Is the rationale for creating the dataset(s) clearly described?

Yes

Are the datasets clearly presented in a useable and accessible format?

Partly

Are the protocols appropriate and is the work technically sound?

Partly

Reviewer Expertise:

bioinformatics and sponge-microbe symbiosis

We confirm that we have read this submission and believe that we have an appropriate level of expertise to confirm that it is of an acceptable scientific standard, however we have significant reservations, as outlined above.

# Reviewer report: Reviewer response for version 1

**DOI:** 10.21956/wellcomeopenres.26645.r124362

**Published:** 2025-06-09

**Authors:** Shikai Liu

**Affiliations:** 1 Ocean University of China, Qingdao, China

In this work, Maldonado et al presented a genome assembly from an individual Crambe crambe, with genome sequence of 143.2 Mb in span. The assmbly is scaffolded into 18 pseudochromomes. A size of 19.53 Kb mitogenome was also assembled for this species. Additionally, several symbiotic prokaryotic genomes were also assembled as MAGs. The sequencing was performed with PacBio HiFi incombination with Hi-C data, which is a well-recognized whole genome sequencing strategy. The data was well presented and should be valuable to the research community.

Are sufficient details of methods and materials provided to allow replication by others?

Yes

Is the rationale for creating the dataset(s) clearly described?

Yes

Are the datasets clearly presented in a useable and accessible format?

Yes

Are the protocols appropriate and is the work technically sound?

Yes

Reviewer Expertise:

Aquatic animal genomics and genetic breeding

I confirm that I have read this submission and believe that I have an appropriate level of expertise to confirm that it is of an acceptable scientific standard.
